# Yeast Mitochondrial Biogenesis: A Role for the PUF RNA-Binding Protein Puf3p in mRNA Localization

**DOI:** 10.1371/journal.pone.0002293

**Published:** 2008-06-04

**Authors:** Yann Saint-Georges, Mathilde Garcia, Thierry Delaveau, Laurent Jourdren, Stephane Le Crom, Sophie Lemoine, Veronique Tanty, Frederic Devaux, Claude Jacq

**Affiliations:** 1 Laboratoire de Génétique Moléculaire CNRS-UMR8541, Ecole Normale Supérieure, Paris, France; 2 Plateforme Transcriptome IFR36, Ecole Normale Supérieure, Paris, France; Wellcome Trust Sanger Institute, United Kingdom

## Abstract

The asymmetric localization of mRNA plays an important role in coordinating posttranscriptional events in eukaryotic cells. We investigated the peripheral mitochondrial localization of nuclear-encoded mRNAs (MLR) in various conditions in which the mRNA binding protein context and the translation efficiency were altered. We identified Puf3p, a Pumilio family RNA-binding protein, as the first trans-acting factor controlling the MLR phenomenon. This allowed the characterization of two classes of genes whose mRNAs are translated to the vicinity of mitochondria. Class I mRNAs (256 genes) have a Puf3p binding motif in their 3'UTR region and many of them have their MLR properties deeply affected by *PUF3* deletion. Conversely, mutations in the Puf3p binding motif alter the mitochondrial localization of *BCS1* mRNA. Class II mRNAs (224 genes) have no Puf3p binding site and their asymmetric localization is not affected by the absence of *PUF3*. In agreement with a co-translational import process, we observed that the presence of puromycin loosens the interactions between most of the MLR-mRNAs and mitochondria. Unexpectedly, cycloheximide, supposed to solidify translational complexes, turned out to destabilize a class of mRNA-mitochondria interactions. Classes I and II mRNAs, which are therefore transported to the mitochondria through different pathways, correlated with different functional modules. Indeed, Class I genes code principally for the assembly factors of respiratory chain complexes and the mitochondrial translation machinery (ribosomes and translation regulators). Class II genes encode proteins of the respiratory chain or proteins involved in metabolic pathways. Thus, MLR, which is intimately linked to translation control, and the activity of mRNA-binding proteins like Puf3p, may provide the conditions for a fine spatiotemporal control of mitochondrial protein import and mitochondrial protein complex assembly. This work therefore provides new openings for the global study of mitochondria biogenesis.

## Introduction

The crucial role of mitochondria in eukaryotic cells is reflected, at least in part, by the increasing number of nuclear genes encoding mitochondrial proteins implicated in human diseases [1]. Mitochondria are complex structures; yeast mitochondria may contain up to 1000 different proteins, accounting for about 17% of the proteins synthesized in this simple cell [2] [3]. Tight spatial and temporal control of the production of these proteins is clearly required to achieve the multiple mitochondrial functions required to respond to the diverse demands of cells. Many studies have addressed this question, using genetic and biochemical approaches to focus on a few mitochondrial complexes. For example, respiratory complex IV (RCC4) has been shown to be assembled via highly sophisticated regulatory loops, with the production of mRNAs and proteins from 33 nuclear and mitochondrial genes [4]. The assembly of respiratory complexes, reflecting 2.5 billion years of evolution in eukaryotic cells, is the archetype for the biogenesis of complex cellular structures. Studies of the biogenesis of mitochondrial building blocks require the classification of mitochondrial functional modules and the determination of the spatiotemporal rules governing their establishment. Genome-wide gene expression studies and analyses of physical interactions between proteins and mutant phenotypes have led to the identification of 164 functionally distinct modules containing two or more proteins and 93 modules containing only one protein [5].

Transcript localization and translational regulation provide a means for the posttranscriptional spatial and temporal regulation of protein production and, possibly, complex assembly. It is therefore of prime importance to consider the site of translation of the various proteins comprising the mitochondrial functional modules. The dual genetic origin of the proteins present in mitochondria implies the involvement of two major cellular compartments in translation, as no mRNA has ever been shown to be imported into mitochondria. The subunits encoded by mtDNA are synthesized by the transcriptional/translational machinery present in the mitochondrial matrix. The system developed by Fox [6] made it possible to analyze the *cis* and *trans* elements controlling the mitochondrial translation process. Most mitochondrial genes require several translation regulators; these proteins, located at the membrane, facilitate the co-translational insertion of newly synthesized hydrophobic proteins [6] [7]. Proteins encoded by nuclear genes are translated on cytoplasmic ribosomes and must be transported into and across mitochondrial membranes. Two transport mechanisms have been proposed, both based on a well-characterized machinery of receptors and translocators [9]. Posttranslational processing is consistent with the observation that many proteins generated *in vitro* can be imported into mitochondria. However, since the pioneering work of Butow *et al.* [8] [9] [10] strong experimental evidence has been obtained to suggest that cytosolic ribosomes bound to mitochondria may control a co-translational import process. This process has been studied with microarrays to identify all the nuclear-encoded mRNAs co-purified with mitochondria. Surprisingly, about half of the mRNAs encoding mitochondrial proteins were found to be translated in the vicinity of the mitochondria; the mRNA molecules of this class were named MLRs (mitochondrially-localized mRNAs) [11]. Further analyses focusing on respiratory and translocator complexes provided support for the hypotheses that MLR proteins, the mitochondrial import of which is probably co-translational, are: i) mainly of prokaryotic origin, ii) linked to the first steps of the complex core construction, iii) imported principally via the TOM-TIM23 pathway [12].

The close relationship between mRNA localization and translation is not well understood. However, it has often been suggested that one of the major biological functions of mRNA localization is to restrict the production of the encoded protein to the appropriate region, implying that mRNA is not translated during the translocation process. A family of translational regulatory proteins—the PUF proteins—repress the translation of their target mRNAs by binding to elements located in the 3'UTR in diverse eukaryotic species [13]. One of the best-known example is the yeast protein Puf6p, which blocks the translation of *ASH1* mRNA, localized specifically to the yeast bud, by binding to its 3'UTR [14]. Another one, Puf3p is a multifunctional mRNA binding protein, located in mitochondria, that interacts with the machinery for mitochondrial motility and inheritance [15]. Puf3p is also known to bind to a consensus motif in the 3'UTR of many mRNAs encoding mitochondrial proteins [16] [17]. There is also evidence that the deletion of *PUF3* results in a decrease in mRNA deadenylation and a doubling of the half-life of *COX17* mRNA [18] . Thus, Puf3p proteins probably control the transport/translation/stability of a specific set of mRNAs, as a function of growth conditions, in mitochondrial biogenesis.

We previously carried out genome-wide analyses to identify nuclear-encoded mRNAs which, in wild type standard conditions, are translated to the vicinity of mitochondria. This allowed us to establish an MLR value (Mitochondrial Localization of mRNA) for most of the yeast genes [11]. This new study takes advantage of both important improvements in the microarray technology and a new procedure for calculation of MLR rate which takes into account the contamination level of mitochondrial fraction ([19] and Doc. S1). In addition, we examined the influence of different parameters on the MLR values such as absence or presence of cycloheximide and puromycin and, most importantly, the role of Puf3p protein. This made it possible to group nuclear-encoded proteins which are imported into mitochondria. The MLR values of the most important group of mRNAs (class I) are clearly dependent on the presence of Puf3p. Thus, Puf3p is the first *trans*-acting factor guiding the localization to the vicinity of mitochondria of well-defined functional classes of mRNAs to be identified. Puf3p-dependent mRNAs were mostly found to encode proteins of the mitochondrial translation machinery and involved in translation control and proteins controlling the assembly of respiratory chain complexes; all these proteins play a key role in the early steps of mitochondrial biogenesis. Another group of MLR-mRNAs was found to be Puf3p-independent and to encode structural proteins of respiratory complexes or metabolic pathways. This classification may provide insight into the spatiotemporal program of mitochondrial biogenesis. Also, given the high level of conservation of mitochondrial components across eukaryotes, we expect these results to have a significant impact on our understanding of mitochondrial biogenesis in mammals.

## Results

### A class of mitochondrial localized mRNA putatively bound to Puf3p

**Figure 1:**
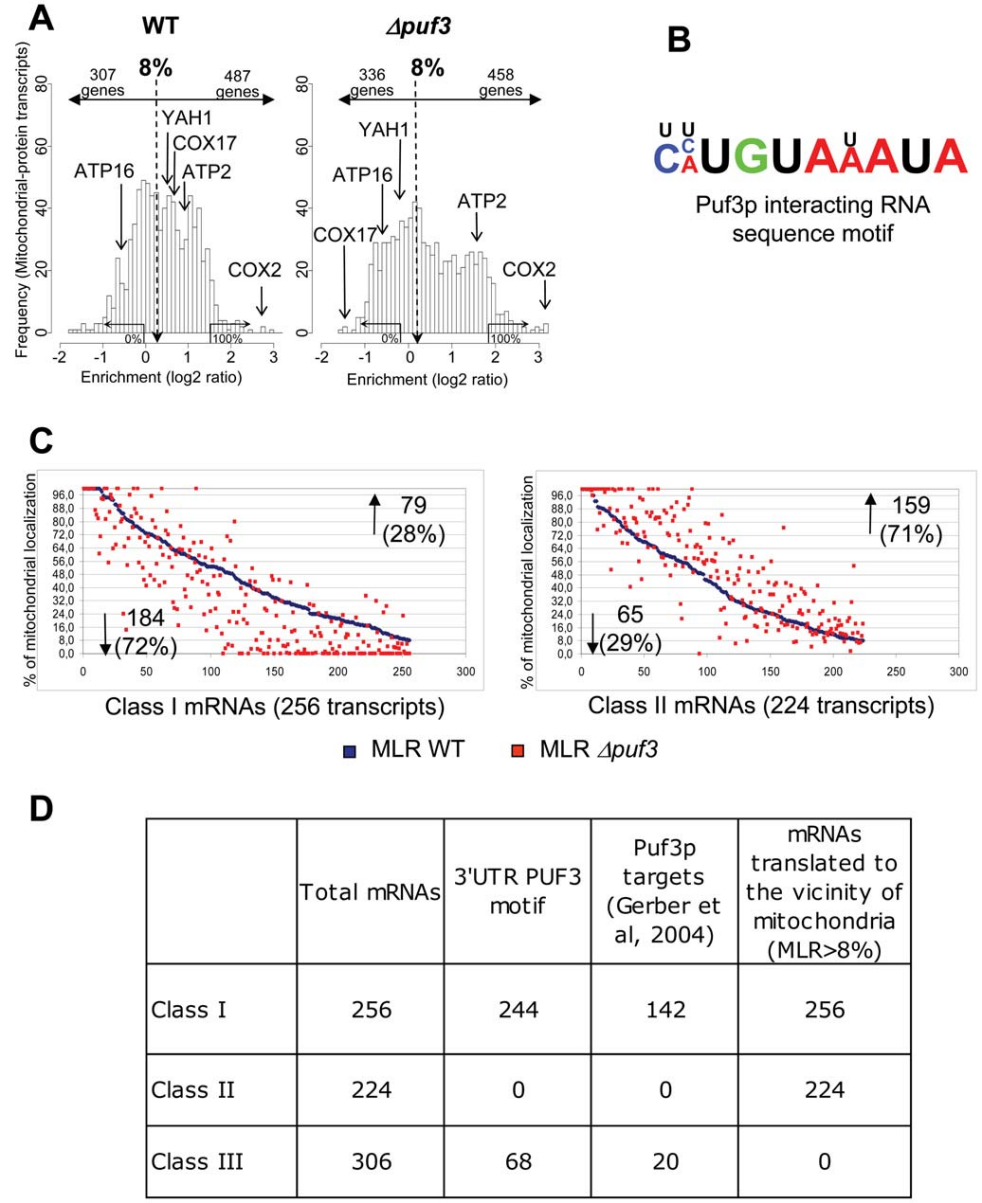
PUF3 deletion affects the asymmetric localization of a specific subclass of MLR-mRNAs A: Distribution of mRNA enrichment in mitochondria preparations for the 794 genes (786 nuclear+8 mitochondrial genes) encoding mitochondrial proteins. The MLR values are expressed as percentages of mitochondrion-bound mRNA fraction, which are derived from the log2 values (see Materials and Methods). A cutoff value of 8% was statistically determined (see Figure S1), which is consistent with the quantitative assessments by RT-PCR of the localization of 100 mRNAs [12]. The number of genes with significantly asymmetrically localized mRNA is indicated on the right of the 8% threshold value. The 100% arrow indicates the median of the log(2) ratio of the 33 mitochondrial transcripts and represent 100% of mitochondrial localization. *COX2* is indicated as a permanent mitochondrial resident (gene and mRNA). The 0% arrow indicates the median of the log(2) ratio of the 416 mRNAs not connected with mitochondria and represents the 0% of mitochondrial localization. The specific case of *COX17* mRNA is worth considering as this mRNA is clearly delocalized in the absence of Puf3p. B: *PUF3* motif : Puf3p target mRNAs(Class I) were defined as having a *PUF3* motif in their 3'UTR and/or as interacting with Puf3p [17]. C: Scatter plot representation of the global asymmetric localization of class I (left panel) and class II (right panel) MLR transcripts in wild-type and Δ*puf3* strains. The set of mRNAs has been plotted in x-coordinate and sorted according to WT mitochondrial percentage of localization from 100% to 8 %, represented by blue squares. For each single mRNA, the corresponding percentage of localization in *Δpuf3* mutant is represented by a red square. The number and percentage of class transcripts showing a decrease or an increase of the mitochondrial localization rate in absence of Puf3p compared to WT is indicated. A significant decrease of the mitochondrial localization rate is observed in absence of Puf3p for the class I MLR mRNAs (p value, p = 3.06 10^−5^). Conversely a significant increase of the mitochondrial localization rate is observed in absence of Puf3p for the class II MLR mRNAs (p value, p = 5.56 10^−3^) D: Table recapitulating the different classes of MLR mRNAs (Class I and II) and non MLR mRNAs (class III). The table shows that the presence of *3'UTR PUF3* motif and/or biochemical association with Puf3p (Puf3p targets column) are predominantly associated with MLR mRNAs (class I+class II) (student test p = 8.4 10^−7^).

**Figure 2:**
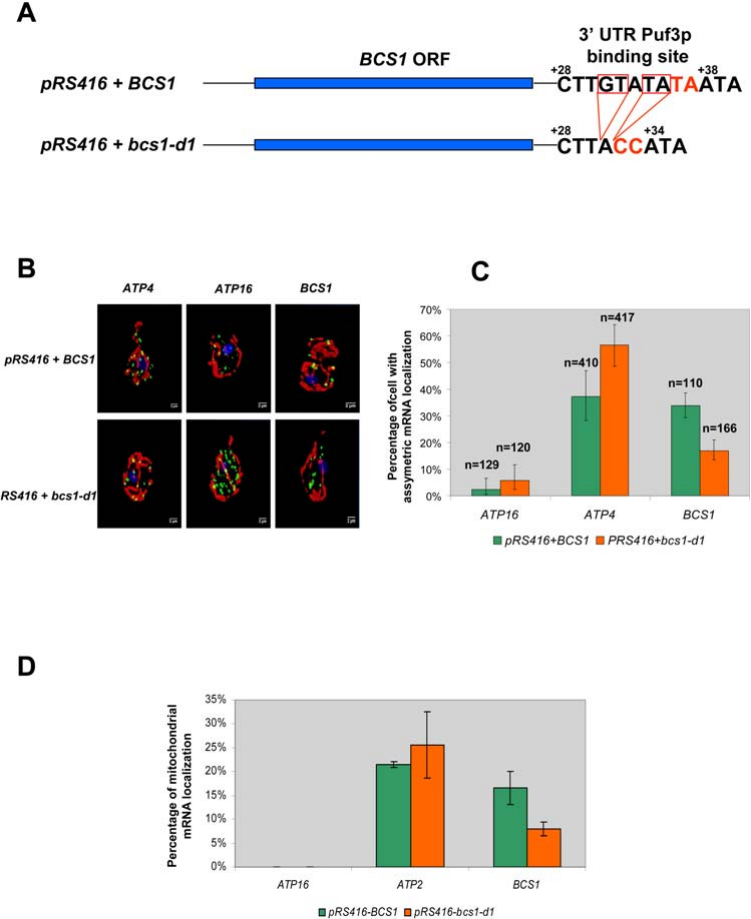
Mutation of the BCS1 Puf3p binding site affects its mRNA mitochondrial localization. A: Six of the ten nucleotides of the putative *BCS1* Puf3p binding site have been deleted (red frame) or substituted (red letters) on pRS416 centromeric plasmid carrying *BCS1* ORF and its own promoter (A generous gift of G. Dujardin). A *bcs1* deleted strain has been transformed by either the plasmid carrying the wild type *BCS1* gene (pRS416+*BCS1*) or a plasmid containing the mutated *bcs1* allele (pRS416+*bcs1-d1*). B: FISH analysis of strain carrying WT or mutated *BCS1* plasmids. Five fluorescent probes specific for mitochondrial ribosomal RNA delimit the mitochondrial compartment (red). Specific mRNAs (*ATP4*, *ATP16*, *BCS1*) were labeled with specific sets of probes (green). The nucleus is stained by DAPI (blue). A total number of 410 cells (*BCS1*) and 417 cells (*bcs1-d1*) were examined using *BCS1* specific FISH probes. At least 100 cells were examined for the *ATP16* and *ATP4* specific probes. Projections of 3D reconstructions are presented for the mutant and wild type cells and the 3 different FISH probes. C: Quantification of the mitochondria/mRNA colocalization rate from the FISH experiment has been done using Corsen software (see Material and Methods) and presented in this histogram. The number of cell used for quantification is presented on top of each bar. Corsen allows the calculation of the percentage of cells showing significant mRNA asymmetric localization to the vicinity of mitochondria. Calculation was done in cells expressing WT (*pRS416+BCS1*) or mutant (*pRS416+bcs1-d1*) *BSC1* alleles for *ATP4*, *ATP16* and *BCS1* mRNAs. For each measure a confident interval is calculated based on a binomial distribution. D: RT-quantitative PCR quantification of mRNAs associated with mitochondrial fraction in cells expressing WT (*pRS416+BCS1*) or mutant (*pRS416+bcs1-d1*) *BCS1* alleles. Total and mitochondrial fraction RNAs were extracted as in Material and Methods. The real time quantification is done according to Garcia *et al.*(12). Each measure was made in triplicate and two independent quantitative PCR have been performed allowing the calculation of a standard deviation presented on the histogram.

Puf3p is an mRNA-binding protein that i) is localized to the cytosolic face of the mitochondrial outer membrane [15], ii) can control translation by speeding up deadenylation [18] and iii) can regulate mRNA stability in response to environmental conditions [20]. Furthermore, using a protein A-tagged Puf3p protein captured with IgG-Sepharose to conduct affinity chromatography, Gerber and colleagues characterized 220 target mRNAs that mainly encode mitochondrially-localized proteins [17]. We therefore addressed the potential role of Puf3p in the MLR process. We carried out comparative global analyses of the mRNAs co-purified with mitochondrial fractions in the presence or absence of the gene *PUF3*, using DNA microarrays. The protocol for global data analyses is described in the “Materials and Methods” section. It takes into account the unavoidable contaminations and provides, for each mRNA species in the cell, a quantitative assessment of the percentage of molecules which are localized to the vicinity of mitochondria. We found that a mitochondrial localization rate of 8% was statistically valid as a cut-off to distinguish between MLR and non-MLR-mRNAs (see Methods, Figure S1). The complete data are available in our website (http://www.biologie.ens.fr/lgmgml/publication/MitoGenesis/) but this work will only consider the 794 genes (including 8 mitochondrially-encoded genes, Table S1), which were considered as coding for mitochondrial proteins. Among these 794 genes, we were able to distinguish 480 nuclear-encoded MLR-mRNAs (Figure 1, Table S1). These 480 nuclear-encoded MLR-mRNAs can be divided in two classes. Class I (256 mRNAs) includes Puf3p targets identified by Gerber et al [17] which code for mitochondrial products (142 mRNAs) plus mRNAs which have a 3'UTR Puf3 motif (114 mRNAs). Class II (224 mRNAs) have no 3'UTR Puf3 motif. Figure 1D summarizes the origins of the different mRNA classes. These genome-wide approaches based on biochemical purifications had to be confirmed by *in situ* analyses. FISH analyses were thus conducted on four mRNAs: 2 class I mRNAs: *YAH1* (MLR = 24%) and *BCS1* (MLR = 31%), 1 class II mRNA: *ATP4* (MLR = 32%) and, finally, 1 class III mRNA (not asymmetrically localized) *ATP16* (Figure S2). Since it is an extremely difficult task to estimate distances between mitochondria and a specific mRNA from 2D representations, we recently developed a software (Jourdren *et al.*, in preparation) to quantify these distances on a large number of cells. We represent the data as the percentage of cells having a significant (see Materials and Methods) mRNA symmetrical distribution. This cell percentage, which supports our mRNA classification, is in agreement (see Figure 2) with quantitative RT-PCR analyses of biochemical purifications. Notably, this quantitative FISH approach underscores the difficulty to conclude from 2D representation of FISH analyses. Thus, in addition to statistical analyses presented above, three-dimensional representations are also provided in the supplemental data (Supplemental data, Movies S1 [Supplementary-material pone.0002293.s006] to S3).

### Role of Puf3p in the asymmetric localization of mRNA

The effect of *PUF3* deletion on the mitochondrial association of the whole set of MLR transcripts was not statistically significant (p = 0.24) compare to wild type control. However, the effect of this deletion on class I MLR transcripts was significant (p = 3.06 10^−5^). Indeed, 72% of the mRNAs have their MLR value decreased in Δ*puf3* strain (Figure 1C). Many of them, like *COX17* mRNA, are completely delocalized when *PUF3* is deleted (Figure 1A). *COX17* mRNA deserves a special mention since several studies have focused on its interaction with Puf3p. *COX17* mRNA has been shown to interact directly with Puf3p and it was observed that *PUF3* deletion leads to an increase in the amount of *COX17* mRNA [18]. The fact that Puf3p could be involved in both mRNA stability and mRNA localization prompted us to look for relationship between the two processes. We thus analyzed the stationary level of 11 different mRNAs in the presence or absence of *PUF3*. The increase in mRNA level connected to deletion of *PUF3* seems to be linked to a decrease in mitochondrial localization of the mRNA (Figure S3). Interestingly, when *PUF3* is deleted, *COX17* mRNA and *COX 23* mRNA are 5.2 and 3.3 fold more abundant respectively and they are no longer asymmetrically localized (MLR near 0). These two mRNAs have two puf3p binding sites in their 3'UTR and are thus more dependent on Puf3p for both stability and localization. More mRNAs should be examined for their half-life in the wild type and Δ*puf3* contexts to firmly confirm this negative correlation between mRNA localization and abundance and better define the role of *PUF3* in this relationship.

We also wondered whether this Puf3 effect on mRNA localization depends on direct interactions with the corresponding mRNA. To address this point we analyzed the MLR properties of a mutated form of the putative Puf3p binding motif of *BCS1* mRNA. *BCS1* is interesting since it is a class I mRNA with a MLR value of 31%. In man, mutations in BCS1L gene are responsible for pathologies with various clinical presentations *e.g.* GRACILE syndrome [21]. Figure 2 presents experimental evidence that deletion of Puf3p binding motif alters the localization properties of *BCS1* mRNA. Quantitative FISH analyses (Figure 2C) show a significant decrease in the percentage of cell which present an asymmetric distribution of the mutated form of *BCS1* mRNA. This is in full agreement with the Q-PCR analyses of the accumulation of the *BCS1* transcript in the mitochondrial fraction, a totally independent biochemical approach (Figure 2D). Finally, the fact the MLR values of class II mRNAs, which, by definition, have no Puf3 motif, were not affected by *PUF3* deletion and remained firmly associated with mitochondria (Figures 1A, 2C, 2D and table S1) is another argument in favor of a direct connection between Puf3p and class I mRNAs.

The fact that Puf3p is also probably involved in the regulation of mRNA-specific rates of translation prompted us to study the role of translation inhibitors in the mRNAs physical interaction with mitochondria.

### Effects of the translation inhibitors cycloheximide and puromycin on mRNA localization

**Figure 3:**
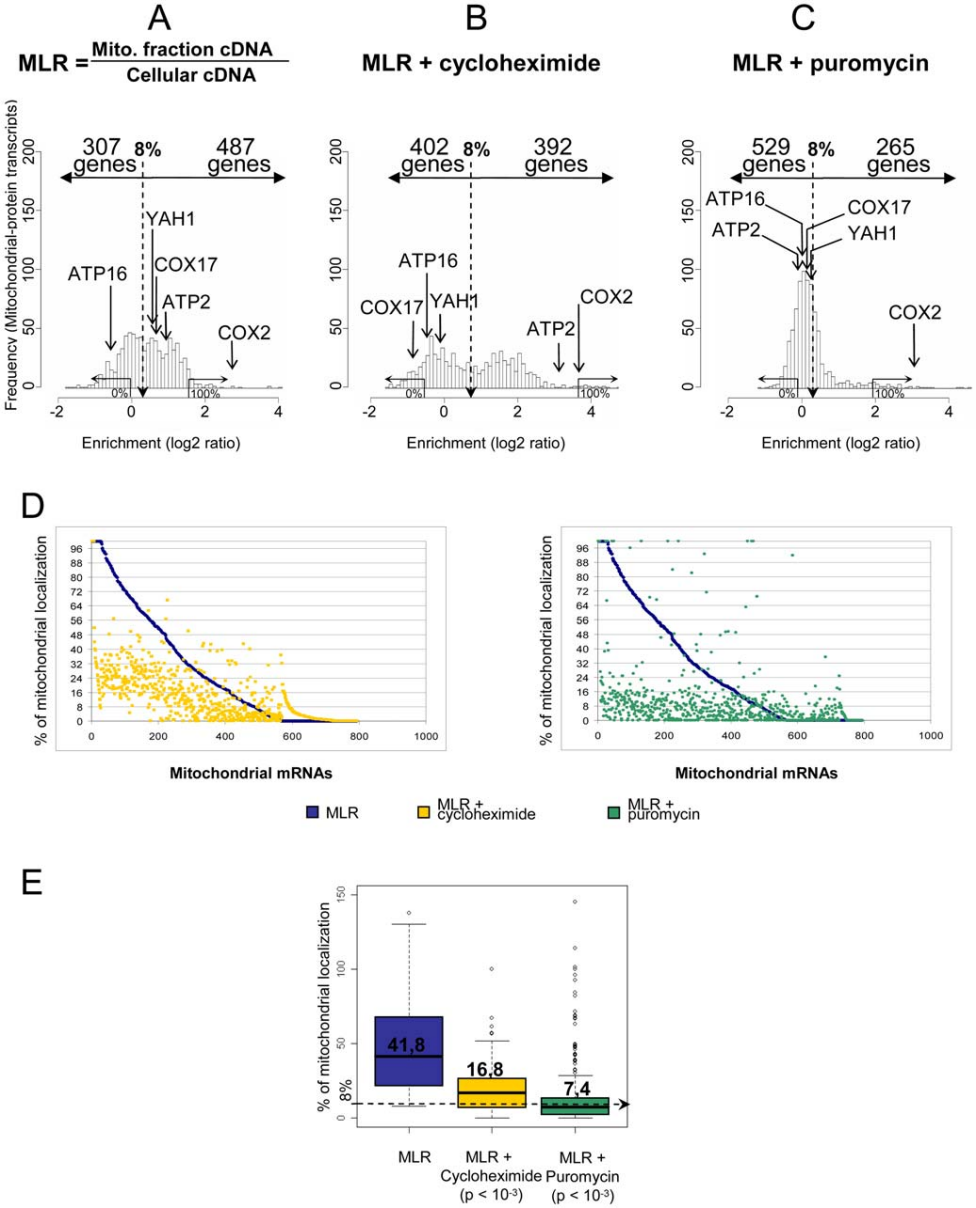
Effects of cycloheximide and puromycin on the asymmetric localization (MLR) of nuclear-encoded mitochondrion-linked mRNAs. The distributions focus on the 794 genes encoding mitochondrially localized proteins (A) in presence of cycloheximide (B) or puromycin (C). The threshold of 8% was determined as in figure 1. A few mRNAs are indicated as markers of different subcellular translation sites: mitochondrial mRNAs (*COX2*), free cytoplasmic polysomes (*ATP16*), mitochondrion-bound cytoplasmic mRNAs delocalized to free polysomes by cycloheximide (*COX17, YAH1*), or mitochondrion-bound mRNA whose mitochondrial enrichment is increased by cycloheximide (*ATP2*). All represent different classes of mRNAs, the properties of which are described in supplementary Table S1. D: Scatter plot representation of the Mitochondrial Localization Rate of the 794 transcripts extracted from cells treated in presence of cycloheximide (left panel) or puromycin (right panel) and compared to untreated cells. The set of mRNAs has been plotted in x-coordinate and sorted according to the untreated cell mitochondrial percentage of localization (MLR) and represented by blue squares. For each mRNA, the corresponding percentage of localization calculated from extract of cell treated with translation inhibitor is represented by a yellow square (cycloheximide) or green square (puromycin). E: Box-plot representation of the asymmetric localization of mRNAs encoding mitochondrial proteins. The line through the center of each box represents the median value of the distribution; the size of the box represents the median absolute deviation of the distribution. Three sets of experimental conditions were analyzed in triplicate: no translation inhibitor (left), with cycloheximide (centre), with puromycin (right).

The action of puromycin was studied in high-salt conditions, which have been shown to stimulate the release of the ribosomes from mitochondria [10]. This protocol was derived from studies of membrane-bound ribosomes in secretory cells, in which ribosomes are released from microsomes following puromycin treatment, in a medium of high ionic strength [22]. Our finding (Figures 3C, 3D, 3E, Supportive Information Figure S4 and Table S1) that 71% of the mRNAs have their MLR value which is dramatically reduced in the presence of puromycin+KCl suggests that ribosome association is required to maintain the asymmetric localization of these mRNAs. The mitochondrial association of the other 29% mRNAs may be more dependent on direct mRNA-mitochondria interactions. More experiments are necessary to clarify this interesting point which may reveal subgroups of mRNAs.

**Figure 4:**
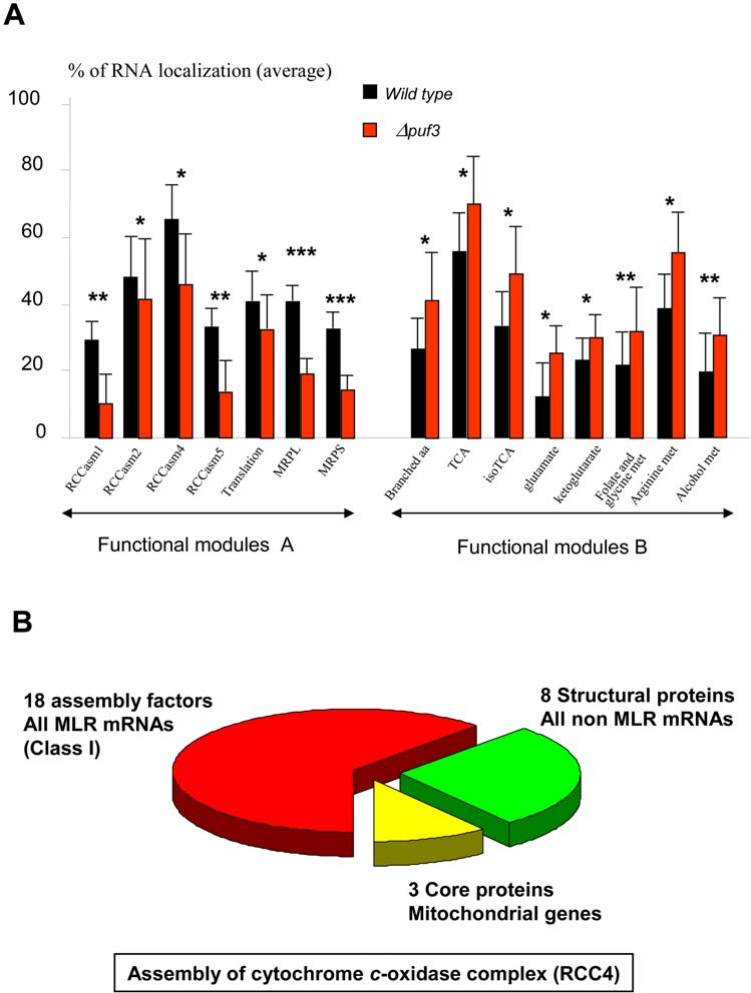
PUF3 deletion affects the asymmetric localization of different functional modules differently. A: Distribution of *Δpuf3* effects on some of the mitochondrial functional modules described by Perocchi [5]. Only the functional modules for which average MLR value was significantly affected in the *Δpuf3*strain have been represented. The mean RNA localization value of each functional module is either decreased (class A) or increased (class B). Asterisks denote the p values (Student test) of each wild type *versus* mutant differences: *  = p<0.05, **  = p<0.01, ***  = p<0.001. All A-type functional modules are involved in translation or are assembly factors: thus RCCasm modules (1 to 5) include 29 genes (*ATP11, ATP12, SHY1, COX17*, etc…); translation module includes 15 genes mainly involved in mitochondrial translation control (*CBS2, CBP6, PET494*, etc…); MRPL and MRPS contain 48 and 37 genes coding for mitochondrial ribosomal proteins respectively ; B-type functional modules concern metabolic pathways: Branched aa (10 genes), TCA (13 genes), isoTCA (6 genes), Glutamate (6 genes), Ketoglutarate (13 genes), Folate (10 genes), Arginine (5 genes), Alcohol (10 genes). Detailed for all these modules can be found in [5] and in Table S1. The significant increased mean MLR value after *PUF3* deletion might be the consequence of the modification of the global PUF proteins due to the absence of Puf3p. B. The specific example of cytochrome c-oxidase complex biogenesis (RCC4). Concerning the nuclear encoded subunits, the 18 assembly factors necessary for the synthesis of the complex [4] are translated on polysomes linked to mitochondria whereas the 8 structural proteins are translated on cytoplasmically-free polysomes (Table S4 for gene details).

**Figure 5:**
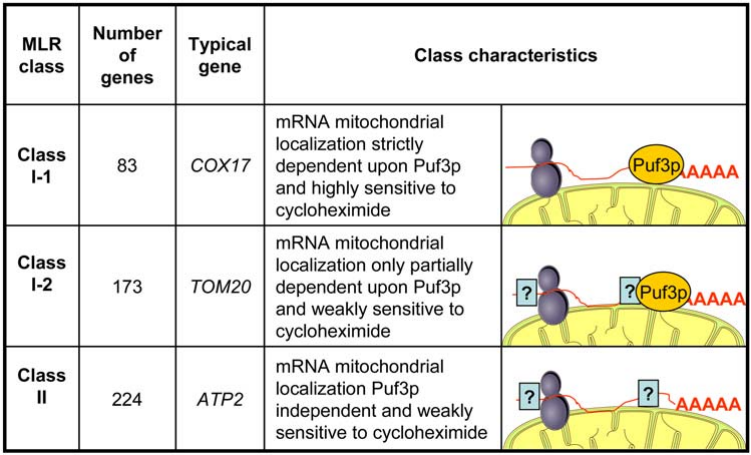
The 480 mRNAs localized to the vicinity of mitochondria (MLR-RNAs) can be classified according to their Puf3p-dependence. Two main classes of MLR-mRNAs can be identified: class I and class II are distinguished by the presence or absence of at least one *PUF3* motif in their 3'UTR sequence respectively. In addition classI mRNAs can be, in the absence of *PUF3*, totally (class I-1) or partially (class I-2) delocalized. This property seems to be correlated with the high sensitivity to cycloheximide for class I-1 mRNAs. We propose that class I-1 Puf3p target mRNAs requires mainly Puf3p for their mitochondrial localization whereas class I-2 mRNAs localization also depends on other unknown trans-acting products. Class II mRNAs are localized to the vicinity of mitochondria under the control of unidentified trans-acting factors.

The widespread belief that cycloheximide, which blocks the translocation reaction on cytoplasmic ribosomes, selectively stabilizes the mRNAs-ribosomes associations initially led researchers to treat the cells with cycloheximide before the extraction of membrane-bound polysomes [9] [23] [11]. We nevertheless carried out comparative microarray analyses to assess the impact of cycloheximide on the MLR process. To our surprise (Figure 3, B and D), cycloheximide treatment increased or decreased the levels of mitochondrion-bound mRNAs. A box-plot representation (Figure 3E) comparing the distributions of the mitochondrial localization rates of MLR-mRNAs in different experimental situations shows that the median value of the distribution, indicated by the line through the center of the box, was significantly decreased by cycloheximide. This global decrease in the asymmetric distribution of mRNAs linked to mitochondria resulted from the cycloheximide-dependent delocalization of 135 mRNAs, while 40 mRNAs were more asymmetrically distributed after cycloheximide treatment (Table S1). This second observation is consistent with the common assumption that the translation inhibitor stabilizes ribosome-mRNA associations. By contrast, the delocalization of 135 mRNA molecules in the presence of cycloheximide was unexpected. These 135 mRNAs (Table S1) correspond principally to genes involved in ribosome assembly and the assembly of respiratory complexes. Remarkably, most of them are targets of Puf3p. This cycloheximide effect is not easily explained. Our recent observation that mitochondrial mRNA localization depends on both cis-acting signals and translation suggests that an active translation process might be required for the asymmetric localization of some mRNAs (Garcia *et al*., in preparation).

### MLR-mRNAs and functional modules

Puf3p was previously shown to target mRNAs involved in specific functions and protein complexes (e.g. the subunits of the mitochondrial ribosome) [17] . Therefore, *PUF3* may have an important role in protein complex assembly, in addition to its role in determining the MLR properties of individual genes. We used the recently published catalog of the mitochondrial functional modules [5] to investigate this aspect. The absence of *PUF3* had two opposite effects on the MLR values of functional modules (Figure 4A). In *Δpuf3* strain, modules involved in translation (ribosomal proteins, translation regulators etc.) and in the assembly of respiratory chain complexes (RCCasm1 to 5) had lower average MLR values whereas metabolic modules (TCA, iTCA, branched amino acids etc.) had higher average MLR values. The first of these situations (Figure 4B, class A modules) is easy to explain because the corresponding modules contain mostly class I genes, which have at least one *PUF3* motif in their 3'UTR. On the other hand, the increase in the MLR value of class B modules may result from an indirect effect due to the absence of *PUF3,* resulting in an imbalance in the mRNA-binding protein populations. This is in agreement with the increased MLR values of 71% of class II mRNAs in *Δpuf3* strain (Figure 1C). Thus, the combinatorial action of PUF RNA-binding proteins on different mRNA classes may be destabilized by the absence of one of these proteins.

These effects of *PUF3* on specific mRNA localization can also be pictured (Figure 4B) for a typical mitochondrial complex such as the respiratory chain complex IV (RCCIV). Significantly 17 out of the 18 known assembly factors required for the biogenesis of this complex [4] are encoded by class I MLR-mRNAs clearly depending on *PUF3*. This all the more striking since the 8 other nuclear genes coding for structural proteins have their mRNA translated on free cytoplasmic polysomes (Figure 4B and Table S3).

## Discussion

Among 786 nuclear genes coding for mitochondrial proteins we characterized two classes of mRNAs translated to the vicinity of mitochondria. Class I (256 genes, Table S 1) and class II (224 genes, Table S 1) genes connected or not connected to Puf3p respectively; class III (306 nuclear genes) mRNAs are translated on free cytoplasmic polysomes.

### A role for Puf3p in the early steps of mitochondria biogenesis

In yeast, the overall importance of Puf3p for mitochondria has been highlighted by genome-wide studies, which have identified 220 putative Puf3p-associated transcripts [17]. We provide the first experimental evidence for a connection between Puf3p and the asymmetric localization of 256 mRNAs, which are translated, close to mitochondria, into proteins subsequently imported into this organelle. This role of Puf3p in the spatial segregation of a well-defined class of mRNAs is supported by the recent observation that Puf3p is actually localized to the outer mitochondrial surface [15].

Puf3p is likely to control the early steps of mitochondria biogenesis. This Puf3p property is especially clear for the assembly factor modules recently defined [5]. These modules—RCCasm1 to 5—are composed of 24 proteins required for the construction of respiratory complexes, but they do not form an integral part of these complexes. For instance, cytochrome c-oxidase assembly (COX, RCC4) is a complicated process requiring 18 assembly factors for the correct assembly of 12 structural proteins [4]. Remarkably, all the 18 assembly factors are translated in the vicinity of mitochondria, and 17 out of 18 of these MLR-mRNAs serve as targets for Puf3p (Figure 4B, Table S3). In this respect, the fact that the transcriptional regulation of nuclear COX assembly genes by carbon source differs from that of nuclear COX structural genes [4] is especially interesting. In particular, the expression of some of these assembly genes, including *COX10* and *COX17,* is little affected by the diauxic shift and the promoter regions of these genes have no Hap target sequence in agreement with an absence of transcriptional regulation. We propose that the Puf3p-MLR process, which is involved in the production of assembly factors but not structural proteins, is a good candidate for the specific regulation of this aspect of COX biogenesis. Accordingly, Puf3p should be highly regulated. Puf3p steady-state levels are known to decline during the diauxic shift [15].

Such observations in yeast are probably relevant to other eukaryotic cells in which the MLR process occurs [24] and in which COX assembly factors [25], for instance, may play a role in human diseases. In mice and in human cancer cells, COX assembly factors Sco2p, which yeast ortholog clearly produces an MLR-mRNA, are targeted by p53, potentially accounting for the effects of p53 on metabolism and aging [26].

Thus, Puf3p identifies class I MLR-mRNAs, with translation products playing a fundamental role in the very early steps of mitochondrial biogenesis, and anchors them to the surface of mitochondria. Puf3p also increases mitochondrial motility [15], thereby ensuring the faithful transfer of mitochondria from mother to daughter cells and the early stages of mitochondrial biogenesis, by dragging a specific class of mRNAs into the daughter cell.

### Puf3p, translation, localization and degradation of mRNAs

In many eukaryotic cells, the PUF proteins bind to the 3′ untranslated region of mRNA and regulate transcript translation and/or decay in a carbon source-dependent manner [13], [20]. We demonstrate here a new function for this class of protein: specific mRNA localization. The molecular mechanisms that lead to the coordination between localization, translation and degradation of mRNAs are almost completely unknown even if there are suggestive indications of a strong connection between regulation of translation and mRNA asymmetric sorting [27]. Puf3p may play a role all along these processes. Certainly Puf3p is not the only protein to be involved and other proteins might modulate its different activities. It was previously observed [17] [28] [29] that several PUF proteins can control a single mRNA. We observed (Figure 5) that Puf3p-dependent mRNA localization can concern two sub-groups of class I genes whose mRNA localization exhibit different cycloheximide sensitivity and degree of Puf3p dependence, suggesting that other unidentified proteins might be involved. On the other hand, the specific localization of the 224 class II mRNA (Figure 5 and table S1), which is *PUF3*-independent, should rely on other RNA-binding proteins and some candidates are being studied.

Finally, our data strongly support the “posttranscriptional RNA regulon” model, according to which eukaryotes regulate related mRNAs according to their functional organization into ribonucleoprotein (RNP) complexes [30]. Genome-wide studies revealed distinct programs of RNA regulation orchestrated by specific RNA-binding proteins and/or non-coding RNAs [31]. We show here that Puf3p is certainly one of the key proteins of class I ribonucleoprotein complexes involved in the early steps of mitochondria biogenesis. This is a static description of a highly dynamic organization. As recently observed in mammalian cells, the time-controlled translation of critical mitochondrial constituents in the vicinity of mitochondria is linked to mitochondrial dynamics during the cell cycle [24]. Also, in continuous yeast culture systems, which spontaneously generate metabolic cycles, the most periodic genes are class I Puf3p-dependent genes [32] . This strongly suggests that the production and the localization of class I mRNAs are closely coordinated. These observations highlight the importance of Puf3p in mitochondrial biogenesis and segregation.

## Materials and Methods

### Strains and plasmids used

All the strains used in this study are isogenic to CW252, an intronless mitochondrial genome with a nucleus isogenic to that of W303 [33] with the exception of the *BCS1* Puf3p binding site mutation studies. In this latter experiments a BY4742, *bcs1::kanMX4* (Euroscarf) was used for FISH and Q-PCR analyses. The *puf3Δ* strain was constructed by homologous recombination, using a PCR-amplified DNA fragment from the *puf3* (*YLL013c*) locus of the strain BY4741, *YLL013c::kanMX4* (Euroscarf). The pRS416+*BCS1* plasmid is a gift from G. Dujardin and was used for mutagenesis of the Puf3p binding sequence motif. Direct mutagenesis was performed using QuikChange^R^ II Site-Directed Mutagenesis Kit with these two oligonucleotids:

BCS1delpuf3for:

GCTAAGTGGCGCCTTACTACATAACTT**ACC**ATAAAAATTCAGAAG

BCS1delpuf3rev:

CTTCTGAATTTTTATGGTAAGTTATGTAGTAAGGCGCCACTTAGC

### Extraction of mitochondrion-associated RNA

Mitochondrion-associated RNA and total cellular RNA were extracted as previously described [19]. Cells were grown at 30°C in galactose medium (1% bactopeptone, 1% yeast extract, 2% galactose, 0.1% KH_2_PO_4_ and 0.12% (NH_4_)_2_SO_4_). Cells were incubated for 10 minutes and fractionated, in the presence or absence of 200 µg/ml cycloheximide. Cells were treated with puromycin to release mitochondrion-bound cytoplamic ribosomes. The cells were fractionated in the presence of 2.1 mM puromycin and 1 M KCl as previously described [10]. RNA was extracted and purified from the various fractions using the RNEasy Mini Kit (Qiagen). Three independent RNA preparations (mitochondrial+cellular RNA) were obtained in the absence of drugs and two independent preparations were obtained for each treatment with cycloheximide or puromycin and in the absence of Puf3p. The quality of the extraction was checked by quantitative PCR analysis as previously described [19].

### Array hybridization and normalization

The microarray data and the related protocols are available at the GEO web site (www.ncbi.nlm.nih.gov/geo/), accession number: GSE9393. Briefly, the cellular and mitochondrion-associated RNAs of each independent preparation were reverse-transcribed and labeled with Cy3 or Cy5 dye using the indirect labeling procedure. We then hybridized 1.5 µg of cellular and mitochondrial labeled cDNA with ab 8×15K *S. cerevisiae* DNA chip manufactured by Agilent. Each array hybridization was replicated using dye-swap. Arrays were read using a GenePix 4000B scanner (Molecular Devices) and the signal segmentation was done using the GenePix Pro 6.1 software. Data pretreatment was applied on each result file to discard GenePix flag and saturating spots. The data were normalized without background substraction by the global Lowess method performed with the Goulphar software [34].

### Percentage of mRNA asymmetric localization and SAM analysis

In RT-PCR analyses, the percentage of each mRNA localized in mitochondrial fraction was calculated in a manner similar to that used in a previous study [19]. For the calculation of the mitochondrial localization rate from microarray data, we used a method adapted from [19]. A detailed protocol is presented in Doc S1. Briefly, we estimated the mitochondrial purification yield from the median log2 values of the mitochondrial fraction/total cell extract ratios of 33 RNAs transcribed from the mitochondrial genome (8 mRNAs, 2 rRNAs and 23 tRNAs). This median value was set at 100% and served as the normalization factor used to transform all the log2 values into mitochondrial localization rates. These raw rates were corrected to take into account the biochemical contamination due to the non-mitochondrial fraction. This task is difficult because the mitochondrial fraction is always contaminated by membrane fractions [23]. However, as negative control, we considered 416 mRNAs not connected with mitochondria (according to YPD, see supplemental table S4) and calculated a median contamination value, which was substracted from the raw localization rates as described in [19] to obtain the final corrected and normalized mitochondria localization rates (MLR values) for each mRNA probed on the array. To generate a list of mRNAs which were statistically enriched in the mitochondrial fraction in our experiments, the microarray data were analyzed with the SAM (significance analyses of microarrays) method implemented in the TMEV software (www.tm4.org), with default parameters and a false discovery rate of 5% [35]. This list was used to set a MLR value cutoff with a reasonable rate of false positive. We observed that a cutoff of 8% led to 3% of mislocalized mRNA according to the list generated by SAM (Figure S1), with a total false positive rate of 8% (5% of SAM FDR plus 3% of discrepancies between SAM and the MLR value cutoff). Therefore, among the 489 MLR-mRNAs identified in this study, only 40 may be false positives. RT-PCR analyses were simultaneously performed on mRNA preparation used for microarrays to validate the method (See Supplemental Data, Table S2). The complete microarray data are available in our website (http://www.biologie.ens.fr/lgmgml/publication/MitoGenesis/).

### FISH analysis and in situ mRNA localization

WT cells were grown on galactose medium (1% bactopeptone, 1% yeast extract, 2% galactose) to mid-exponential growth phase and were treated as described in a previous study [12]. The combined hybridization of three to five antisense oligonucleotides probes was used to enhance the visualization of each mRNA. Typically, each probe was designed such that it contained 50 to 55 nucleotides, with five aminoallyl thymidines and a coherent Tm value. After synthesis by Eurogentec (San Diego, CA), the probes were directly labeled with Cy3 or Cy5 fluorochromes and hybridized to fixed cells as previously described [19].

### Image analyses

Fluorescent imaging and three-dimensional sampling by multiple-stage microscope acquisition allows collecting data within the whole cell but conclusions driven in the absence of 3D reconstitution are highly speculative. To assess relative RNA localizations we developed a software dedicated to distance measurement between two fluorescent probes and allowing analysis of a large cell population. We could thus quantify the distance between mitochondria, revealed with mitochondrial rRNA probes, and a specific mRNA. This analysis is based on 1) 3D segmentation and extraction of object features (coordinates and intensity). 2) Distance measurement of each mRNA particles to the mitochondrial surface. 3) Statistical analysis of data generated: distances are intensity-weighted and for each cell the distribution of mRNA- mitochondria distance is analysed to extract the percentage of particles in the close vicinity of mitochondria (a threshold of 100 nm was chosen to be consistent with microscope resolution). In these conditions if more than 35% of the studied mRNA is localized to mitochondrial vicinity there are 95% chances that the corresponding cell exhibits mRNA asymmetric localisation (see results for ATP16 mRNA). Data represent fraction of cell population with localized mRNA with confidence intervals calculated using binomial test. Such analyses could be routinely carried out on more than 300 cells. The detailed description of the distance-FISH software, called CORSEN, is in preparation (Jourdren et *al*).

## Supporting Information

Figure S1 Correlation SAM analysis-Mitochondrial localization rate. Histograms of the three classes of transcripts determined after SAM analysis: Localized (enriched in the mitochondrial fraction), Undetermined (not enriched or not reduced in the mitochondrial fraction); Not localized (specifically reduced in the mitochondrial fraction). The top histogram represents the distribution of transcripts with a calculated percentage of mitochondrial localization value (MLR) superior (blue) or inferior (red) to 10%. The bottom histogram represents the same distribution, but with a calculated percentage of mitochondrial localization superior (blue) or inferior (red) to 8%. The number in the bars represents the quantity of transcript that belongs to the different classes with the proportion of these classes on the right side of the bars. It is important to note that almost all the transcripts (96,3%) with a percentage of localization superior to 8% belongs to the “localized” class, showing a high correlation between SAM analysis and the calculated percentage of mitochondrial localization.
(0.60 MB TIF)

Figure S2 FISH images of class I, I, III nuclear-encoded mRNAs coding for mitochondrial proteins. Five fluorescent DNA probes specific for mitochondrial ribosomal RNA delimit the mitochondrial compartment (red). mRNAs were labeled with specific fluorescent probes (green). 71 to 190 cells were examined and quantification is represented on a histogram. The quantification using Corsen software (see Materials and Methods, Jourdren *et al.*, in preparation) allows the calculation of the percentage of cells in which a specific mRNA co-localized with mitochondria. The represented confident interval is calculated assuming a binomial distribution. Each classes of transcripts are represented, *ATP16* mRNAs (class III) are not localized to mitochondria whereas *ATP4* (class II) or *BCS1* and *YAH1* (class I) mRNAs co-localized with mitochondria.
(1.22 MB TIF)

Figure S3 Is mRNA accumulation induced in *Δpuf3* strain correlated with mRNA delocalization? To determine a putative correlation between accumulation of class I mRNA and mitochondrial delocalization due to the absence of Puf3p, variation of steady state level of 11 class I mRNAs is plotted against the variation of the MLR value of these transcript in *Δpuf3* versus wild type strain. Pearson correlation value was calculated and shows a poorly significant correlation between accumulation of mRNA and delocalization of mitochondria with a confidence interval of 5%. In addition, SAM50 mRNA was classified as a class I mRNA after it was found as a Puf3p target in studies of Gerber et al (2004, PloS Biol 2 E79), however, *SAM50* mRNA has no Puf3p motif I its 3'UTR and, in our study, its MLR value is not affected by *PUF3* deletion. Thus *SAM50* may not be a class I mRNA and when removed from the above list, the corresponding Pearson value shows no correlation between mRNA accumulation and mitochondrial delocalization. In conclusion, the half-life of more mRNAs should be analyzed to establish the pivotal role of Puf3p in localization-translation-degradation of all these class I mRNAs.
(0.45 MB TIF)

Figure S4 High salt concentration does not lead to dissociation of mRNAs from the mitochondria. We analyzed by quantitative RT-PCR the amount of 9 mRNAs co-purified with mitochondria in different conditions: 0, 1 M KCl, 1 M KCl+puromycin. As previously observed [10], the salt effect is not sufficient to free the mRNAs from mitochondria; only the double action of KCl+puromycin is efficient on the majority of mRNAs (see Results and Table S1). Each measure was made in triplicate and a minimum of two independent quantitative PCR has been performed allowing the calculation of a standard deviation presented on the histogram.
(0.65 MB TIF)

Movie S1 Colocalization of *ATP4* a class II mRNA with mitochondria in WT cells. The image J plugin Volume Viewer has been used to reconstruct a 3 dimensional view of *ATP4* mRNA localization (green) with mitochondria (red) in the cells pictured in the Figure S2. The nucleus is stained in blue.
(1.38 MB MOV)

Movie S2 *ATP16* a class III mRNA is not localized to the vicinity of mitochondria. The image J plugin Volume Viewer has been used to reconstruct a 3 dimensional view of *ATP16* mRNA localization (green) with mitochondria (red) in the cells pictured in the Figure S2. The nucleus is stained in blue.
(1.24 MB MOV)

Movie S3 *YAH1* a class I mRNA colocalizes with mitochondria in WT cells. The image J plugin Volume Viewer has been used to reconstruct a 3 dimensional view of *YAH1* mRNA localization (green) with mitochondria (red) in the cells pictured in the Figure S2. The nucleus is stained in blue.
(0.93 MB MOV)

Table S1 MLR properties of the mRNAs coding for mitochondrial products. The genes are grouped in functional modules following the work by Steinmetz group (Perocchi F, Jensen LJ, Gagneur J, Ahting U, von Mering C, et al. (2006). PLoS Genet 2: e170). Four MLR classes of RNA are distinguished by different colours. Class I and class II are mRNAs whose statistical analyses (SAM q values) indicate an asymmetrical localization. This mitochondrial localization is dependent or independent of the mRNA binding protein Puf3p, respectively. Class III corresponds to mRNAs translated on cytoplasmically-free ribosomes and class IV are mRNAs encoded in the mitochondrial genome. The MLR values (% of Mitochondrial Localization of mRNA) are represented for the wild type cell in standard growth conditions or with either cycloheximide or puromycin. The mutant strain *Δpuf3* has also been analyzed. The decision whether a gene has prokaryotic homologue is from SGD database. Puf3p binding site is from the data of (Foat BC, Houshmandi SS, Olivas WM, Bussemaker HJ (2005) Proc Natl Acad Sci USA 102: 17675–17680) and (Gerber AP, Herschlag D, Brown PO (2004) PLoS Biol 2: E79). Puf3p target signifies that the corresponding mRNA has been retained in affinity chromatography with Puf3p (Gerber AP, Herschlag D, Brown PO (2004) PLoS Biol 2: E79).
(0.38 MB XLS)

Table S2 RT-PCR analyses of mRNA localization. RT-PCR analyses were conducted as indicated in Garcia M, Darzacq X, Devaux F, Singer R, Jacq C (2007); Biology MiM, editor. Totowa, New Jersey: Humana Press. 505–528 p.
(0.02 MB XLS)

Table S3 The COX assembly factors are class I MLR-mRNAs. The different classes of genes were described previously (Fontanesi F, Soto IC, Horn D, Barrientos A (2006) Am. J. Physiol. Cell 291:C1129–1147). All the assembly factors are translated to the vicinity of mitochondria and 17 out 18 are likely to interact with Puf3p. The 8 nuclearly-encoded mRNAs coding for structural proteins are translated on free cytoplasmic polysomes.
(0.04 MB XLS)

Table S4 List of the genes coding for cytosolic non-mitochondrial proteins (according to YPD) which were used as a negative control set
(0.07 MB XLS)

Doc S1 QUANTITATIVE PCR ANALYSIS OF ASYMMETRIC mRNA LOCALIZATION.
(0.18 MB DOC)
