# Supplementary figures and images for: Yeast Mitochondrial Biogenesis: A Role for the PUF RNA-Binding Protein Puf3p in mRNA Localization
Source: PLoS One. 2008 Jun 4;3(6):e2293. doi: 10.1371/journal.pone.0002293 (PMC2387061; doi:10.1371/journal.pone.0002293)

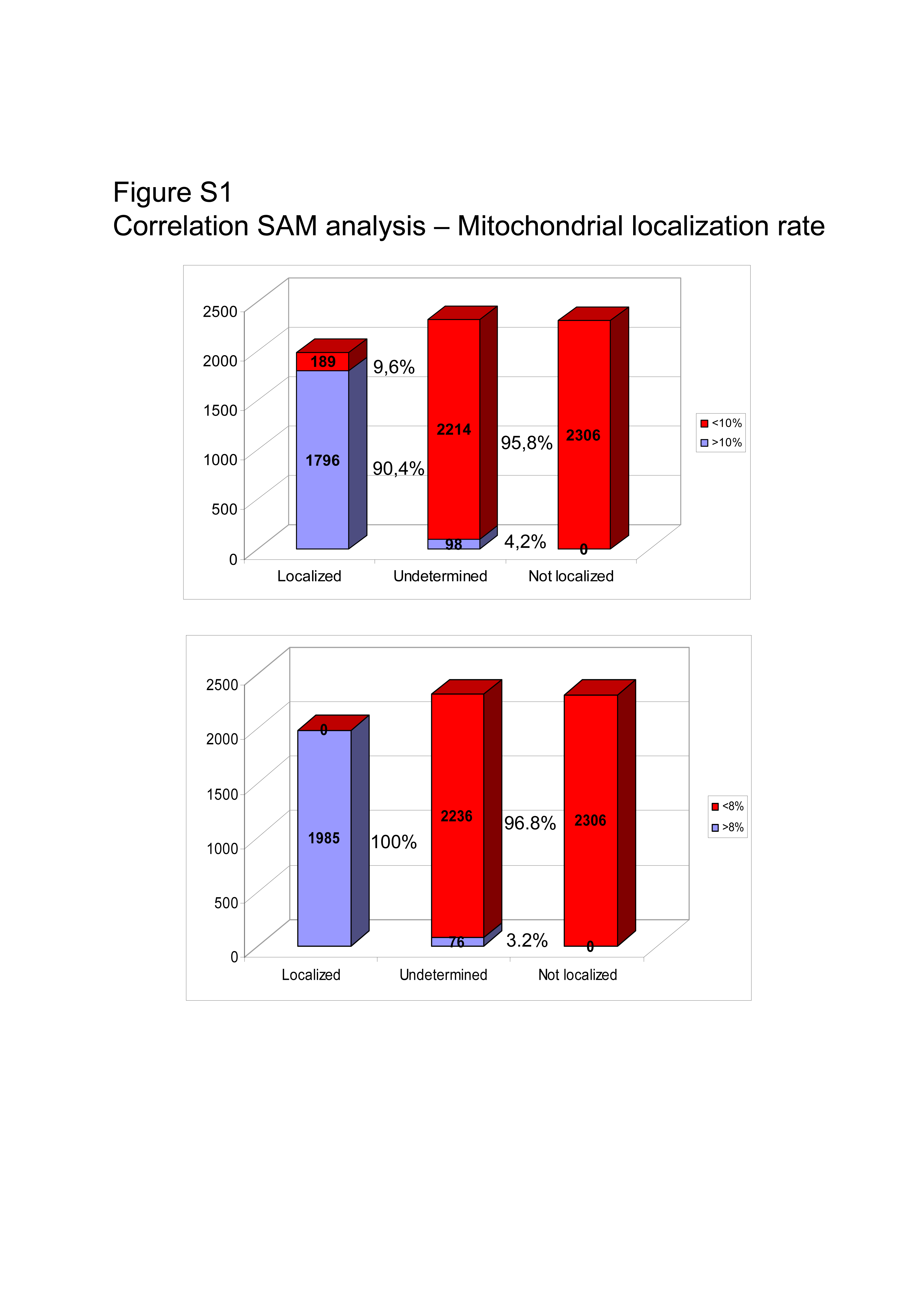

Supplement: Figure S1 — Correlation SAM analysis-Mitochondrial localization rate. Histograms of the three classes of transcripts determined after SAM analysis: Localized (enriched in the mitochondrial fraction), Undetermined (not enriched or not reduced in the mitochondrial fraction); Not localized (specifically reduced in the mitochondrial fraction). The top histogram represents the distribution of transcripts with a calculated percentage of mitochondrial localization value (MLR) superior (blue) or inferior (red) to 10%. The bottom histogram represents the same distribution, but with a calculated percentage of mitochondrial localization superior (blue) or inferior (red) to 8%. The number in the bars represents the quantity of transcript that belongs to the different classes with the proportion of these classes on the right side of the bars. It is important to note that almost all the transcripts (96,3%) with a percentage of localization superior to 8% belongs to the “localized” class, showing a high correlation between SAM analysis and the calculated percentage of mitochondrial localization. (0.60 MB TIF) [file pone.0002293.s001.tif]

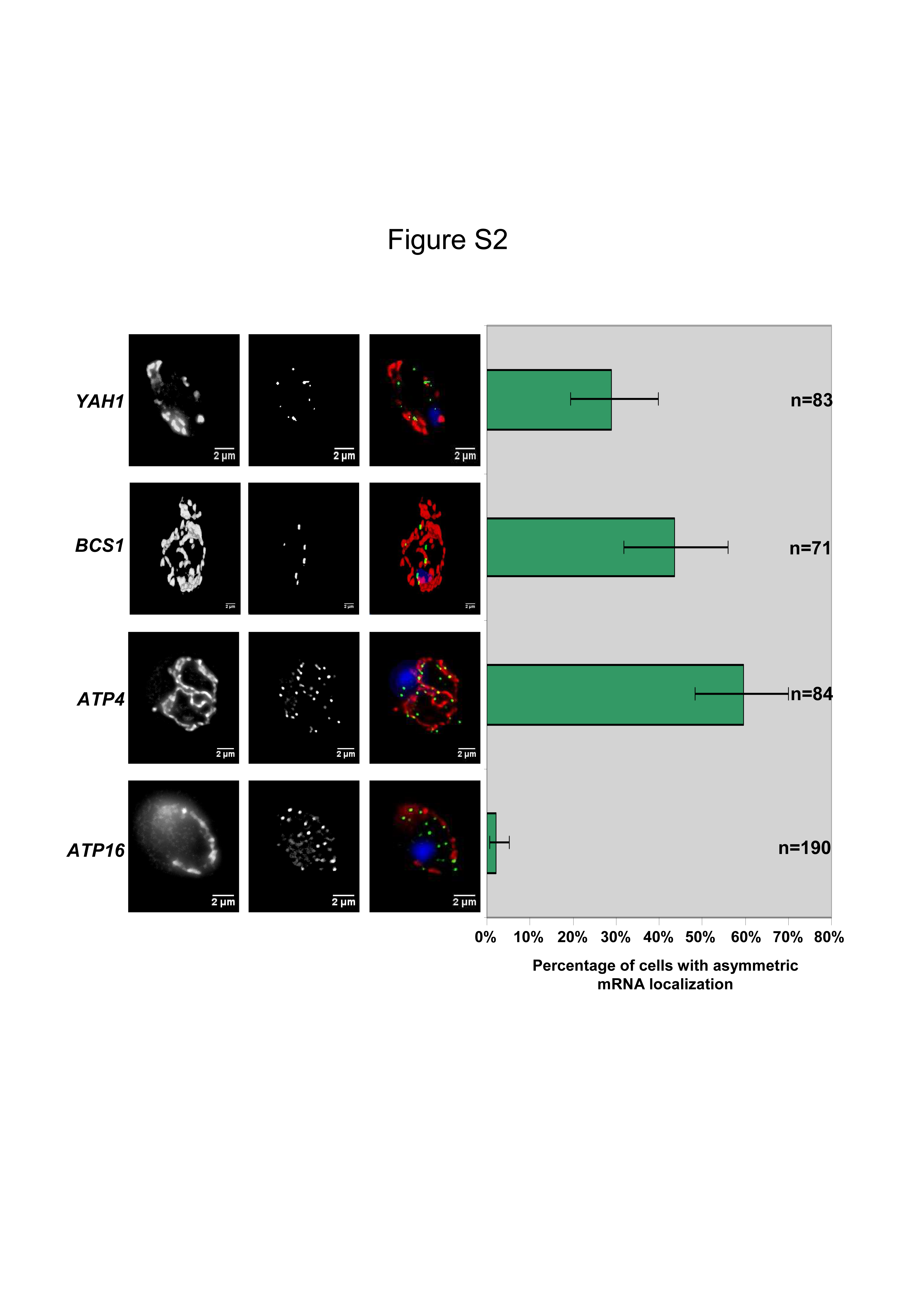

Supplement: Figure S2 — FISH images of class I, I, III nuclear-encoded mRNAs coding for mitochondrial proteins. Five fluorescent DNA probes specific for mitochondrial ribosomal RNA delimit the mitochondrial compartment (red). mRNAs were labeled with specific fluorescent probes (green). 71 to 190 cells were examined and quantification is represented on a histogram. The quantification using Corsen software (see Materials and Methods, Jourdren et al., in preparation) allows the calculation of the percentage of cells in which a specific mRNA co-localized with mitochondria. The represented confident interval is calculated assuming a binomial distribution. Each classes of transcripts are represented, ATP16 mRNAs (class III) are not localized to mitochondria whereas ATP4 (class II) or BCS1 and YAH1 (class I) mRNAs co-localized with mitochondria. (1.22 MB TIF) [file pone.0002293.s002.tif]

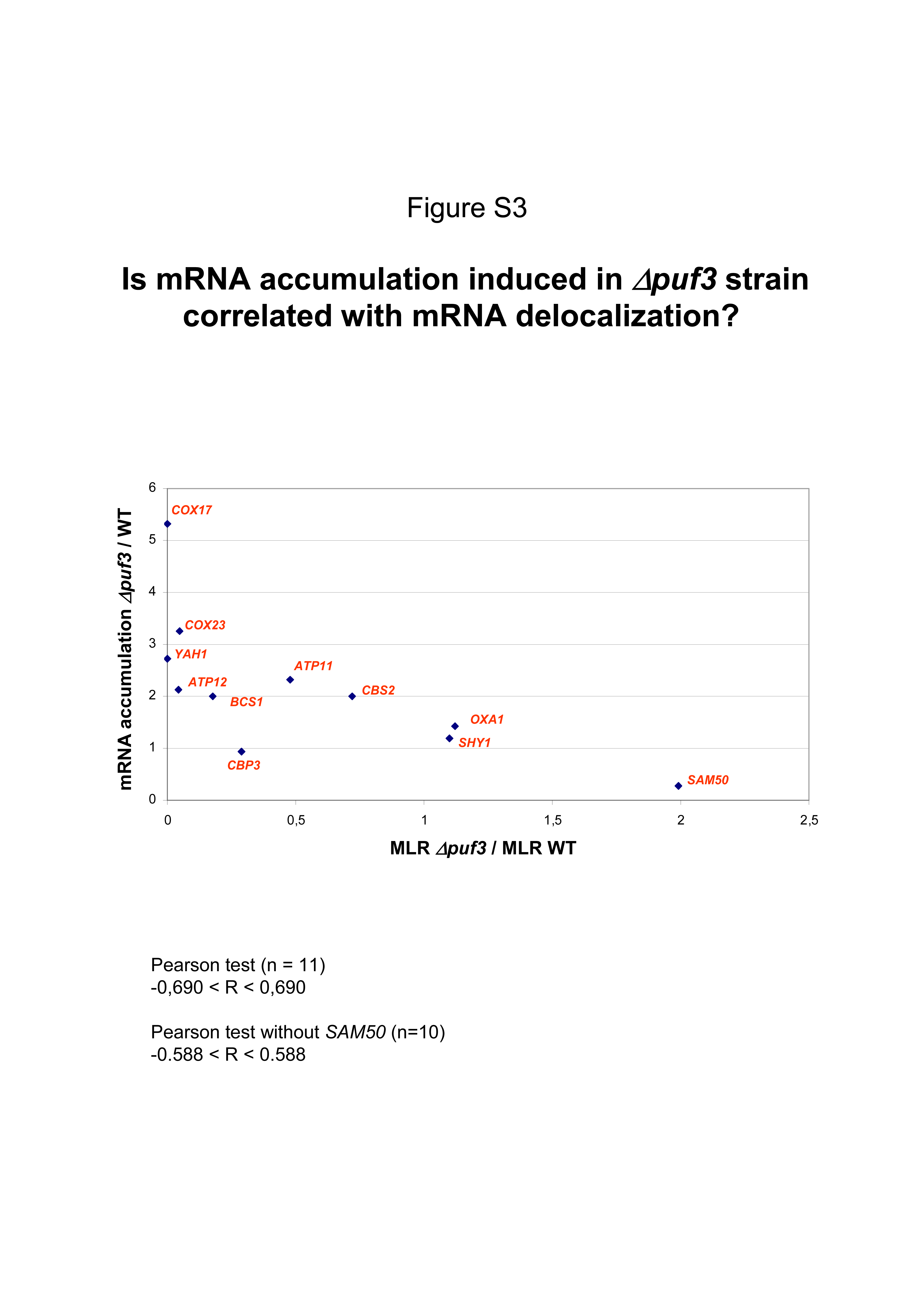

Supplement: Figure S3 — Is mRNA accumulation induced in Δpuf3 strain correlated with mRNA delocalization? To determine a putative correlation between accumulation of class I mRNA and mitochondrial delocalization due to the absence of Puf3p, variation of steady state level of 11 class I mRNAs is plotted against the variation of the MLR value of these transcript in Δpuf3 versus wild type strain. Pearson correlation value was calculated and shows a poorly significant correlation between accumulation of mRNA and delocalization of mitochondria with a confidence interval of 5%. In addition, SAM50 mRNA was classified as a class I mRNA after it was found as a Puf3p target in studies of Gerber et al (2004, PloS Biol 2 E79), however, SAM50 mRNA has no Puf3p motif I its 3'UTR and, in our study, its MLR value is not affected by PUF3 deletion. Thus SAM50 may not be a class I mRNA and when removed from the above list, the corresponding Pearson value shows no correlation between mRNA accumulation and mitochondrial delocalization. In conclusion, the half-life of more mRNAs should be analyzed to establish the pivotal role of Puf3p in localization-translation-degradation of all these class I mRNAs. (0.45 MB TIF) [file pone.0002293.s003.tif]

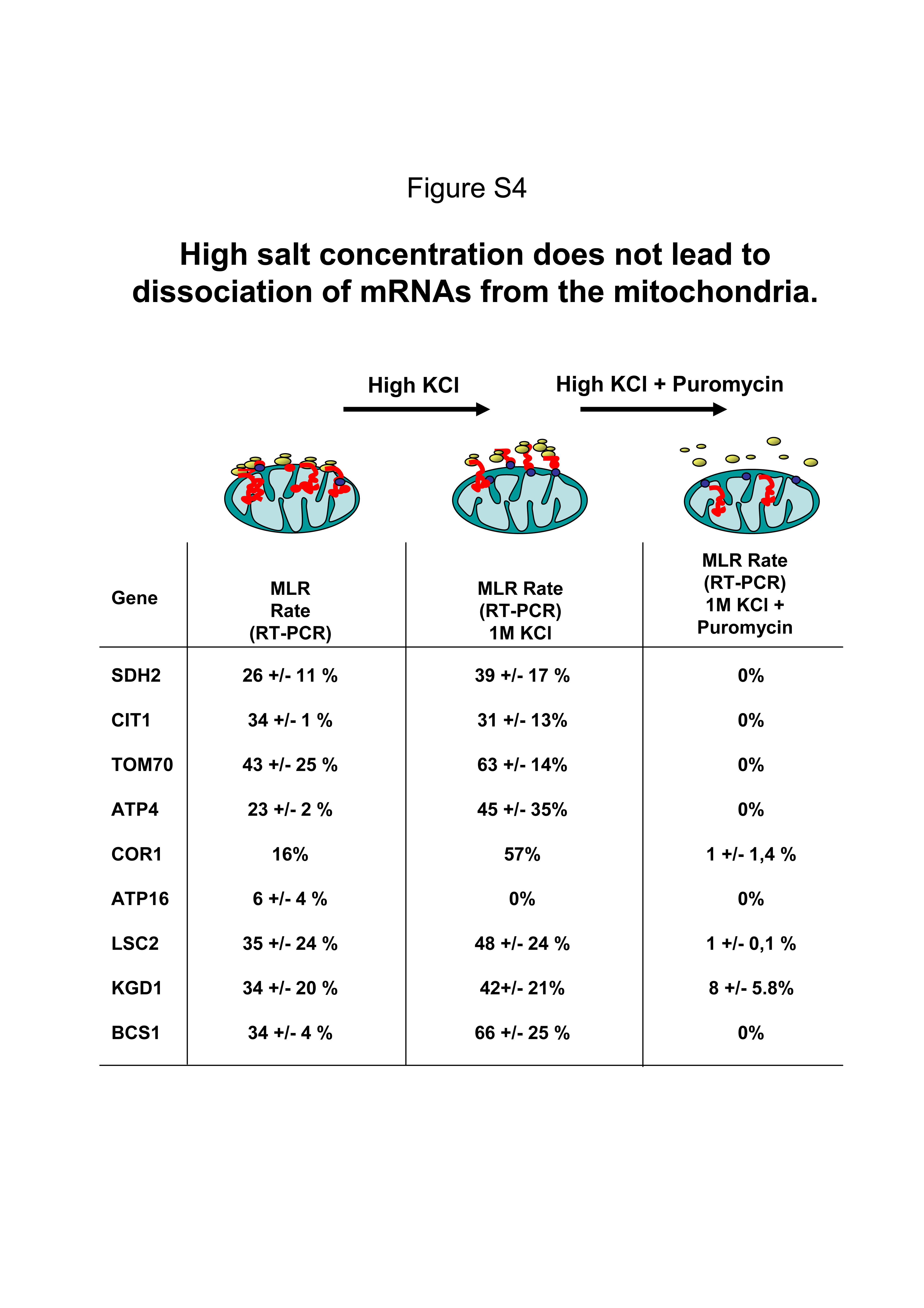

Supplement: Figure S4 — High salt concentration does not lead to dissociation of mRNAs from the mitochondria. We analyzed by quantitative RT-PCR the amount of 9 mRNAs co-purified with mitochondria in different conditions: 0, 1 M KCl, 1 M KCl+puromycin. As previously observed [10], the salt effect is not sufficient to free the mRNAs from mitochondria; only the double action of KCl+puromycin is efficient on the majority of mRNAs (see Results and Table S1). Each measure was made in triplicate and a minimum of two independent quantitative PCR has been performed allowing the calculation of a standard deviation presented on the histogram. (0.65 MB TIF) [file pone.0002293.s004.tif]
