# Supplementary material for: Yeast Mitochondrial Biogenesis: A Role for the PUF RNA-Binding Protein Puf3p in mRNA Localization
Source: PLoS One. 2008 Jun 4;3(6):e2293. doi: 10.1371/journal.pone.0002293 (PMC2387061; doi:10.1371/journal.pone.0002293)
Supplement: Doc S1 — QUANTITATIVE PCR ANALYSIS OF ASYMMETRIC mRNA LOCALIZATION. (0.18 MB DOC) [file pone.0002293.s012.doc]

Doc. S1: QUANTITATIVE PCR ANALYSIS OF ASYMMETRIC mRNA LOCALIZATION. A complete version of this protocol has been published in Garcia M, Darzacq X, Devaux F, Singer R, Jacq C (2007); Biology MiM, editor. Totowa, New Jersey: Humana Press. 505-528 p

The strain CW252 isogenic to W303 should be favoured due to its intron-less mitochondrial genome allowing an easier detection of mitochondrial transcripts.

- 1. Mitochondria bound and total RNA isolation for Q-PCR and microarrays
     1. YPGal: 1% (w/v) bactopeptone, 1% (w/v) bacto yeast extract, 2% (w/v) galactose. Autoclave 30 min at 110°C. Store at room temperature.
     2. Gal-rich medium: 1% (w/v) bactopeptone, 1% (w/v) bacto yeast extract, 2% (w/v) galactose, 0.1% (w/v) KH_2_PO_4_, 0.12% (w/v) (NH_4_)_2_SO_4_. 1 ml mix is made and dispensed in two 2 ml erlenmeyer flasks and then autoclaved for 30 min at 110°C.
     3. Pre-incubation buffer: 100 mM Tris-HCl, pH 9.3, 0.5 M β-mercaptoethanol. Do not store: should be prepared just before use.
     4. Digestion buffer: 20 mM potassium phosphate buffer, pH 7.4, 1.35 M sorbitol. Do not store: should be prepared the day before the mitochondria isolation.
     5. Zymolyase 100T (Seikagatu corporation 120493)
     6. Washing medium: 1% (w/v) bactopeptone, 1% (w/v) bacto yeast extract, 1 M sorbitol, 0.1% (w/v) KH_2_PO_4_, 0.12% (w/v) (NH_4_)_2_SO_4_. Autoclave 30 min at 110°C.
     7. Regeneration medium: 1% (w/v) bactopeptone, 1% (w/v) bacto yeast extract, 2% (w/v) galactose, 1M sorbitol, 0.1% (w/v) KH_2_PO_4_, 0.12% (w/v) (NH_4_)_2_SO_4_. Autoclave 30 min at 110°C.
     8. Sorbitol-cycloheximide Ice cube: 1 M sorbitol, 200µg/ml cycloheximide. Should be prepared the day before the mitochondria isolation and stored at –20°C.
     9. Cycloheximide solution: 100 mg/ml cycloheximide in ethanol solvent. Prepare 1 ml the day of mitochondria isolation: store at 4°C before use. Should not be conserved more than one day.
     10. Sorbitol-cycloheximide buffer: Prepare 1 M sorbitol stock solution. Autoclave 30 min at 110°C. Store at room temperature. Just before use add 200 µg/ml cycloheximide to 100 ml 1 M sorbitol.
     11. Mannitol buffer: 0.6 M mannitol, 30 mM Tris-HCl, pH 7.6, 5 mM MgAc, 100 mM KCl. Autoclave 30 min at 110°C. Store at room temperature. Just before use complete with 5 mM β-mercaptoethanol, 200µg/ml cycloheximide, 500 µg/ml heparine, and 1 l for 20 g of yeast dry weight of protease inhibitors (Sigma).
     12. 30 ml Thomas Glass Potter with striated tip for a more efficient cell broke.
  2. RNA purification for quantitative PCR and microarrays
     1. TES buffer: 10 mM Tris-HCl, pH 7.5, 10 mM EDTA, 0.5% (v/v) SDS. Autoclave 30 min at 110°C. Store at room temperature.
     2. Phenol chloroform mix: phenol:chloroform 5:1 (Sigma ref: P-1944). Store at 4°C.
     3. Chloroform: Sigma
     4. Ready Red. Store at 4°C.
     5. Sodium acetate: 3 M NaAc, pH 5.3. Autoclave 30 min at 110°C. Store at room temperature.
     6. RNA purification kit: NucleoSpin RNA II kit from Macherey-Nagel.
     7. Water: Use Molecular Biology Water (Eurobio) for good RNA stability.
  3. Quantitative RT-PCR
     1. PCR machine (mastercycler Eppendorf) is used for incubation steps.
     2. Primers: Random hexamers from Roche (ref :1034731) and Oligo dT from Invitrogen (ref : yo1212). Store at -20°C.
     3. RT Primer mix: 5 µg of random hexamers (2.5µl), 2 µg of oligo dT (4 µl). Complete with RNAse free water to a final volume of 23 µl. Prepare on ice just before use.
     4. BRL Superscript II kit from Gibco: this kit contains 5X SSII Buffer, Superscript enzyme and DTT (0.1 M). Store at –20°C.
     5. dNTP mix: 2.5 mM dATP, 2.5 mM dTTP, 2.5 mM dCTP, 2.5 mM dGTP. Store at -20°C.
     6. Reaction mix: 8 µl of SSII buffer, 4 µl DTT, 2 µl dNTP. Prepare on ice just before use.
     7. PCR extract kit: for retrotranscription products purification use Nucleospin Extract Kit from Macherey-Nagel.
     8. LighCycler instrument from Roche (ref: 2011468).
     9. LightCycler Capillaries (20 µl) from Roche (ref: 1909339).
     10. Barrier tips (neptum) should be used to avoid DNA contamination from pipetman.
     11. QuantiTech Sybr Green PCR kit from QIAGEN (ref: 204143).
     12. Primers: For every target transcript a primer pair (for and rev primers) should be designed following kit instruction. Each primer is dissolved to a final concentration of 100 pmol/µl (10X primer). For and rev primers are then mixed to a final concentration of 10 pmol/µl (Q-PCR primer mix). 10X primers and primer mix are stored at -20°C.Three types of primer pairs are used for precise quantification of RNA transcript localization: target transcript primers are used to quantify RNA of interest in each fraction, normalisation primers are used to quantify mitochondrial RNA (for example: COX1 and COX2) and determine mitochondria purification yield, and contamination marker primers (for example: ACT1 and RPL10) are used to evaluate cytosolic RNA contamination.
  4. Labelled cDNA synthesis for microarray analyses
     1. Mastercycler personal from Eppendorf.
     2. Random hexamers and Oligo dT (12-18) from Invitrogen.
     3. Molecular biology grade water from Eurobio.
     4. Superscript II RNase H reverse transcriptase, RT 5X first strand buffer and 0.1 M DTT from Invitrogen.
     5. Cy3- and Cy5-linked dUTP from Amersham.
     6. dNTP set, 100 mM from Pharmacia.
  5. RNA hydrolysis before cDNA purification
     1. NaOH pure from Prolabo.
     2. HCl 37% from Carlo Erba.
  6. Purification of labelled cDNA for microarray hybridization
     1. 2K15 centrifuge from Sigma.
     2. 5415 D centrifuge from Eppendorf.
     3. Ethanol 95%, spectrophotometer grade from Carlo Erba.
     4. Qiaquick PCR purification kit.
     5. Na Acetate 3M, pH5.2.
  7. Quantitative RT-PCR
     - 1. PCR machine (mastercycler Eppendorf) is used for incubation stepsPrimers: Random hexamers from Roche (ref :1034731) and Oligo dT from Invitrogen (ref : yo1212). Store at -20°C.
       2. RT Primer mix: 5 µg of random hexamers (2.5µl), 2 µg of oligo dT (4 µl). Complete with RNAse free water to a final volume of 23 µl. Prepare on ice just before use.
       3. BRL Superscript II kit from Gibco: this kit contains 5X SSII Buffer, Superscript enzyme and DTT (0.1 M). Store at –20°C.
       4. dNTP mix: 2.5 mM dATP, 2.5 mM dTTP, 2.5 mM dCTP, 2.5 mM dGTP. Store at -20°C.
       5. Reaction mix: 8 µl of SSII buffer, 4 µl DTT, 2 µl dNTP. Prepare on ice just before use.
       6. PCR extract kit: for retrotranscription products purification use Nucleospin Extract Kit from Macherey-Nagel.
       7. LighCycler instrument from Roche (ref: 2011468).
       8. LightCycler Capillaries (20 µl) from Roche (ref: 1909339).
       9. Barrier tips (neptum) should be used to avoid DNA contamination from pipetman.
       10. QuantiTech Sybr Green PCR kit from QIAGEN (ref: 204143).
       11. Primers: For every target transcript a primer pair (for and rev primers) should be designed following kit instruction. Each primer is dissolved to a final concentration of 100 pmol/µl (10X primer). For and rev primers are then mixed to a final concentration of 10 pmol/µl (Q-PCR primer mix). 10X primers and primer mix are stored at -20°C.Three types of primer pairs are used for precise quantification of RNA transcript localization: target transcript primers are used to quantify RNA of interest in each fraction, normalisation primers are used to quantify mitochondrial RNA (for example: COX1 and COX2) and determine mitochondria purification yield, and contamination marker primers (for example: ACT1 and RPL10) are used to evaluate cytosolic RNA contamination.
  8. Mit**o**chondria bound and total RNA isolation for Q-PCR and microarrays

1. Pre-culture: CW252 yeast strain is grown in 20 ml YPGal at 30°C during 24 h with agitation (250 rpm).

2. Culture: an appropriate aliquot of pre-culture is transferred in 1 l Gal-rich medium and incubated for one night at 30°C with agitation: the next morning OD_600nm_ should be between 0.8 and 3 (CW252 generation time in Gal-rich medium is around 2.5 h). The OD_600nm_ must be properly measured before mitochondria isolation in order to evaluate dry weight (DW) using the formula DW= 0.28x OD_600nm_ xV (V: volume in liter).

3. Cells are collected by centrifugation for 15 min at 4300 g at 4°C and washed with water stored at 4°C. Note that all centrifugation steps allowing to wash and change culture medium are performed for 15 min at 4300 g at 4°C.

4. The cells are suspended in 20 ml per g of dry weight of pre-incubation buffer, and then incubated in a 200 ml Erlenmeyer flask at 30°C with agitation for 10 min. Cells are then washed several times with 20 mM potassium phosphate buffer, pH 7.4: a total volume of one liter of buffer is used for this washing step.

5. Cells are suspended in 1 l Erlenmeyer flask to a final OD_600nm_ of 12 in digestion buffer. 5 mg of zymolyase is added to the culture and the mix is incubated for 10 min with agitation for enzymatic digestion of cell walls. After incubation, digestion should be performed at 80%. To verify digestion efficiency, the decrease of OD_600nm_ can be measured by mixing 50µl of culture in 1 ml of water. Vigorously shake before measuring OD_600nm_ to perform cell lysis by osmotic shock in water. After digestion, wash cells with washing medium and then incubate in 200 ml of regeneration medium in 1 l Erlenmeyer flask at 30°C with gentle agitation during 3 hours.

6. During incubation step, prepare cycloheximide solution and weight 20 mg of heparin which will be added to 40 ml of mannitol buffer just before use (see step 9).

7. Add 600 µl of cycloheximide solution to the culture to block translation machinery and incubate at 30°C with agitation for 10 additional min. Set apart 8 ml of culture which will be used to prepare total RNA, dispense them in 2ml Eppendorf tubes and centrifuge at 18,000 g at 4°C for 3 min. Wash pellet with 1 M sorbitol. Store the cell pellet at -80°C.

8. Perform a thermal shock to stop cell metabolism by dispensing culture in a beaker containing sorbitol-cycloheximide ice cubes. From that moment, all steps must be performed at 4°C in a cold room. Centrifuge culture at 4300 g for 15 min at 4°C and wash with 100 ml sorbitol-cycloheximide buffer.

9. Suspend the pellet in 4 ml of mannitol buffer. Cells are broken by twenty strokes in a glass potter, transferred into a 50 ml falcon tube and centrifuged 8 min at 1700 g to remove nucleus. Supernatant is taken off and transferred in a new falcon. The pellet is suspended once more in 3ml mannitol buffer, submitted to twenty strokes in the glass potter, and against centrifuged at 1700 g for 8 min. The supernatant is added to the previous one and a last step of centrifugation allows complete nuclear removal. Supernatant is taken off and transferred into a 15 ml tube that fits in high speed centrifuge adaptors. Centrifugation at 14,600 g for 30 min, leads to a red pellet of mitochondria, which is washed one time with mannitol buffer before being stored at -80°C.

- 1. RNA purification for Quantitative PCR and microarrays
     1. Suspend cells and mitochondrial pellets in 400 µl TES buffer and transfer in 1.5ml Eppendorf tubes. Add 400 µl of phenol chloroform mix and incubate 15 min at 65°C. During incubation step, vortex tubes 30 seconds every 5 min to homogenise. Centrifuge at room temperature for 15 min at 18,000 g.
     2. Transfer the aqueous phase in a new 1.5 ml Eppendorf tube and add 400 µl of phenol chloroform mix. Vortex 3 times for 30 s, centrifuge 15 min at 18,000 g.
     3. Transfer the aqueous phase in a new 1.5 ml Eppendorf tube and add 300 µl of chloroform. Vortex 3 times for 20 seconds, centrifuge 2 min at 18,000 g.
     4. Transfer aqueous phase in tubes containing 30 µl of sodium acetate, add 600 µl of ethanol and incubate 1 hour at -20°C for RNA precipitation. Centrifuge 15 min at 18,000 g at 4°C and resuspend the pellet in 20 µl of RNAse free water.
     5. Quantify RNA by measuring 260 nm absorbance (one absorbance unit corresponds to 40µg/µl RNA): about 250 µg total RNA purification and 50 µg from mitochondria purification are obtained in routine experiments.
     6. Perform a purification step using RNA purification kit. The RNA clean up is an essential step for the quality of the Reverse Transcription. Just follow the recommendations of Macherey-Nagel kit (total RNA preparation from biological fluids section). Elute the column with 60 µl of water a first time, and then re-elute a second time with the same 60 µl.
     7. Quantify purified RNA. For quantitative RT-PCR, adjust concentration to 50 ng/µl. Store RNA at -20°C. When being used, always keep RNA tubes in ice to avoid degradation.
  2. Quantitative RT-PCR
     1. Retrotransciption: For each sample, add 1 µl of RNA to 23 µl of RT primer mix. Incubate for 10 min at 70°C. Put on ice and add 14 µl of reaction mix and 2 µl of Superscript polymerase. Incubate 10 min at 23°C, then 1 h at 42°C. Store at -20°C. It is recommended to perform a control reaction without added RNA in order to verify the absence contamination in RT mix.
     2. Purification: Purification of RT product is an important step for quantitative PCR efficiency. Just follow the recommendations of Macherey-Nagel PCR extract kit (Protocol for direct purification of PCR products). Elute with 50µl of elution buffer, then dilute to 1/10, 1/50 and 1/100 for quantitative PCR.
     3. Real Time Quantitative PCR: For each sample dilution (1/10, 1/50, 1/100), mix in a capillary tube, 10µl of 2X Sibr Mix, 8µl of H_2_O (from QuantiTech Sybr Green PCR kit), 1µl of RT dilution and 1µl of the desired Q-PCR primer mix. Perform a LightCycler program as follows: a first step of initial polymerase activation of 15 min at 95°C, a second step of 55 cycles of amplification which consists in 15 s at 95°C (denaturation step), 30 s at 54°C (annealing step), and 20 s at 72 °C (extension step). Transition rate is 20°C/s. Fluorescence acquisition is performed during extension step. After amplification, cycler performs a melting curve of product by increasing slowly (0.1°C/s) from a low temperature (65°C) to a high temperature (95°C) and measuring the decrease in the fluorescence. This allows to verify the amplification of a unique and specific product during Q-PCR. The programme ends with a cooling step (20°C/s) to reach 40°C. Don’t forget to carry out a Q-PCR with purified RNA diluted to 1/10 as template in order to check the absence of DNA in RNA purifications.

1. Q-PCR Data analysis

Notations:

In notations *c* stands for cellular extract and *m* for mitochondrial extract

: Total volume of yeast culture used to mitochondrial isolation

: Volume used for total RNA preparation

 : Quantity of RNA purified from mitochondria isolation

 : Quantity of RNA purified from cells

 : Quantity of RNA used for quantitative PCR experiment

$Q_{c}^{\text{PCR}}X$: PCR mix Quantification of X RNA in total cellular RNA

$Q_{m}^{\text{PCR}}X$: PCR mix Quantification of X RNA mitochondrial fraction

 $Q_{c}^{T}X$ : Total quantification of X RNA in total cellular RNA

 $Q_{m}^{T}X$ : Total quantification of X RNA in mitochondrial fraction

 $Q_{m}^{T}X$ :Total quantification of X RNA in mitochondrial fraction after correction by contamination rate.

 $Q_{\text{Cy}}^{T}X$ : Total quantification of X RNA in cytoplasm

 $\text{Rm}(X)$ : X RNA spatial distribution: percentage linked to mitochondria

 : Contamination rate

 $\text{Rm}(X)$ : X RNA spatial distribution after correction by contamination rate.

 $E$ : Q-PCR efficiency, ie number of DNA target obtained after a PCR cycle from one target template (0 ≤ *E* ≤ 2)

 $K$ : Threshold for amplified DNA during Q-PCR used to determine the initial template quantity.

 $\text{Cpm}(X)$ : Mitochondrial Threshold Cycle for X RNA is the Q-PCR cycle at which the amplification plots reaches the threshold in mitochondria RNA.

 $\text{Cpc}(X)$ : Cellular Threshold Cycle for X RNA is the Q-PCR cycle at which the amplification plots reaches the threshold in total RNA.

*Q-PCR fluorescence curve analyses.*

The PCR reaction profile can be divided in three steps: an early background phase, an exponential phase and a plateau. During the exponential phase the amplification course is described by the equation: $Q_{n}=Q_{0}\times E^{n}$, where *Q_n_* is the amount of target at cycle n, *Q_0_* is the initial amount of target, and *E* is the Efficiency of amplification.

To compare target initial amount in different samples, a threshold *K* for amplification is set. *Cp* is the corresponding cycle number required to correlate real-time fluorescence curves to initial template concentration according to the equation: .

Different methods are used to determine Cp: for review see Randy Rasmussen paper available at <http://www.idahotec.com/lightcycler_u/lectures/quantification_on_lc.htm>. In the laboratory, we use the Second Derivative Maximum Method in which no human decision is required to help the software to find the exponential portion of the amplification.

*Determination of Q-PCR Efficiency, E, for a given primer mix.*

Equation can be linearized to:

So arranging the form gives the standard curve equation:

Instead of realising an external standard curve, using genomic DNA, results from the different RNA dilutions can be exploited to determine *E*. Considering the initial quantity of template in non diluted RNA, *Q_0(1/1)_*, for each dilution: and standard curve equation can be modified as follows:

So, the slope of curve gives a direct assessment of Q-PCR efficiency for the studied target.

*Determination of mRNA spatial distribution.*

Mitochondrial RNAs (*COX1, COX2*) are used to normalise Q-PCR results and determine mitochondrial purification yield (η). This yield takes into consideration efficiency of different steps from biochemical purification to RT PCR.

Considering the following equations:

and

and

Purification yield is:

This yield is used to determine mitochondrial spatial distribution for each RNA:

Mitochondrial spatial distribution can be corrected considering contamination rate, which is the mitochondrial localization rate of RNA without connection to mitochondria (for example *ACT1*, *RPL10*).

*Correction of contaminations with non mitochondrial fractions.*

Mitochondrial fraction is always contaminated with other cellular fractions. One can take into account these contaminations if one assumes that some mRNAs are nor connected with mitochondria biogenesis like *ACT1* or *RPL10*. Note that other mRNAs may be considered more pertinent to assess contamination level.

Since, cellular RNA arises from cytoplasm and mitochondria contribution and RNA purified in mitochondrial fraction involves specific RNA interaction and contamination from cytoplasm:

This leads to:

And finally the corrected mitochondrial spatial distribution can be expressed as:

Typical value for Mitochondrial spatial distribution before and after correction are presented in figure 1 for *ACT1*, *ATP2*, *ATP3*, *COX4* and *COX6* transcripts (spatial distribution of *ACT1* is considered to represent the contamination rate).
